# Rationale and methods of a randomized controlled trial of immunogenicity, safety and impact on carriage of pneumococcal conjugate and polysaccharide vaccines in infants in Papua New Guinea

**DOI:** 10.1186/s41479-017-0044-z

**Published:** 2017-12-25

**Authors:** Deborah Lehmann, Wendy Kirarock, Anita H. J. van den Biggelaar, Megan Passey, Peter Jacoby, Gerard Saleu, Geraldine Masiria, Birunu Nivio, Andrew Greenhill, Tilda Orami, Jacinta Francis, Rebecca Ford, Lea-Ann Kirkham, Vela Solomon, Peter C. Richmond, William S. Pomat, for the 10v13v PCV trial team, L. Bele, M. Dreyam, A. Elizah, R. Ford, J. Francis, A. Gihigupa, A. Greenhill, S. Javati, J. Kave, W. Kirarock, M. Lai, B. Martin, G. Masiria, A. Michael, L. Moliki, B. Nagepu, M. Nenikuro, B. Nivio, C. Opa, T. Orami, W. Pomat, G. Saleu, P. Siba, V. Solomon, S. Wana, L. Wawae, M. Yoannes, I. Hwaihwanje, T. Korowi, C. Mond, P. Wari, P. Jacoby, D. Lehmann, A. van den Biggelaar, K. Corscadden, C. de Gier, L. Kirkham, T. Rahman, P. Richmond, R. Thornton, M. Passey

**Affiliations:** 1 Telethon Kids Institute, University of Western Australia, 100 Roberts Road, Subiaco, WA 6008 Australia; 2 Papua New Guinea Institute of Medical Research, Homate Street, Goroka, Eastern Highlands Province 441 Papua New Guinea; 3 The University of Sydney, University Centre for Rural Health, School of Public Health, 61 Uralba Street, Lismore, NSW 2480 Australia; 4 School of Applied and Biomedical Sciences, Federation University, Northways Road, Churchill, VIC 3842 Australia; 5 School of Paediatrics and Child Health, University of Western Australia, Roberts Road, Subiaco, WA 6008 Australia

**Keywords:** Pneumococcal, Vaccine, PCV, PPV, Pneumococcal conjugate vaccine, Pneumococcal polysaccharide vaccine, Papua New Guinea, Randomized controlled trial, RCT

## Abstract

**Background:**

Children in third-world settings including Papua New Guinea (PNG) experience early onset of carriage with a broad range of pneumococcal serotypes, resulting in a high incidence of severe pneumococcal disease and deaths in the first 2 years of life. Vaccination trials in high endemicity settings are needed to provide evidence and guidance on optimal strategies to protect children in these settings against pneumococcal infections.

**Methods:**

This report describes the rationale, objectives, methods, study population, follow-up and specimen collection for a vaccination trial conducted in an endemic and logistically challenging setting in PNG. The trial aimed to determine whether currently available pneumococcal conjugate vaccines (PCV) are suitable for use under PNG’s accelerated immunization schedule, and that a schedule including pneumococcal polysaccharide vaccine (PPV) in later infancy is safe and immunogenic in this high-risk population.

**Results:**

This open randomized-controlled trial was conducted between November 2011 and March 2016, enrolling 262 children aged 1 month between November 2011 and April 2014. The participants were randomly allocated (1:1) to receive 10-valent PCV (10vPCV) or 13-valent PCV (13vPCV) in a 1-2-3-month schedule, with further randomization to receive PPV or no PPV at age 9 months, followed by a 1/5^th^ PPV challenge at age 23 months. A total of 1229 blood samples were collected to measure humoral and cellular immune responses and 1238 nasopharyngeal swabs to assess upper respiratory tract colonization and carriage load. Serious adverse events were monitored throughout the study. Of the 262 children enrolled, 87% received 3 doses of PCV, 79% were randomized to receive PPV or no PPV at age 9 months, and 67% completed the study at 24 months of age with appropriate immunization and challenge.

**Conclusion:**

Laboratory testing of the many samples collected during this trial will determine the impact of the different vaccine schedules and formulations on nasopharyngeal carriage, antibody production and function, and immune memory. The final data will inform policy on pneumococcal vaccine schedules in countries with children at high risk of pneumococcal disease by providing direct comparison of an accelerated schedule of 10vPCV and 13vPCV and the potential advantages of PPV following PCV immunization.

**Trial registration:**

ClinicalTrials.gov CTN NCT01619462, retrospectively registered on May 28, 2012

## Background

An estimated 921,000 children die annually from pneumonia, with the majority of deaths occurring in third-world countries [1]. *Streptococcus pneumoniae* (pneumococcus) is the leading cause of pneumonia deaths in children under 5 years, being responsible for approximately 400,000 of these deaths annually [2]. The pneumococcus is also a leading cause of meningitis, septicemia and otitis media. Infants are at greatest risk of dying from pneumococcal disease [1].

In Papua New Guinea (PNG), acute lower respiratory infection (ALRI) is the most common reason for hospitalization and cause of death in children [3, 4] and the burden of invasive pneumococcal disease (IPD) remains high. In a 7-valent pneumococcal conjugate vaccine (7vPCV) trial that we conducted between 2005–2009 in the Asaro Valley, Eastern Highlands Province (the site of this study), the overall incidence rate of ALRI before age 18 months in 109 controls was 1175/1000 person-years; there were 62 episodes of moderate or severe ALRI (incidence rate 439/1000 person-years), 27% of which occurred between 10 and 18 months of age [5]. In the same trial, the estimated incidence rate of IPD in the first year of life was 4762/100,000 per annum (95% CI 1936–9665), approximately 100 times the rates reported in European infants [6]*.* Upper respiratory tract (URT) pneumococcal carriage starts within weeks of birth in PNG (median age of acquisition is 19 days) [7] and is persistent throughout early childhood, with average pneumococcal carriage rates of 70–80% from age 3 months to 5 years [8, 9]. Moreover, the distribution of pneumococcal serotypes in PNG is broad: in our 7vPCV trial more than 60 different pneumococcal serotypes were identified in the URT of infants followed from birth until 18 months of age, of which 53 were identified in the first month of life [8, 9]. Only 43% of pneumococcal carriage isolates among controls who had not received PCV would have been covered by 13vPCV [8].

### Vaccines for pneumococcal disease

Protection against IPD is by circulating opsonophagocytic IgG antibodies binding to capsular polysaccharides, which determine serotype. More than 90 different pneumococcal serotypes have been identified to date. The recognition that immunity correlated with anti-capsular antibodies led to the development of multivalent polysaccharide vaccines [10, 11]. However, polysaccharides are T-cell independent antigens and therefore the pneumococcal polysaccharide vaccine (PPV) is poorly immunogenic in infants and does not induce immune memory [12, 13]. Pneumococcal conjugate vaccines (PCV) containing polysaccharide antigens of individual serotypes covalently bound to protein carriers, hence transforming polysaccharides into T-cell dependent antigens, became available in 2000. In contrast to PPV, PCVs induce immunological memory and serum antibodies with a strong opsonophagocytic capacity in infants [14, 15] and they are effective in preventing vaccine serotype-associated IPD, pneumonia and mortality in high-risk infants [16–19]. Furthermore, PCVs reduce URT carriage of vaccine serotypes in vaccinated children, thus interrupting circulation of vaccine serotypes in the community, which in turn reduces IPD incidence in unvaccinated individuals through herd immunity [20].

The Global Alliance for Vaccines and Immunization (Gavi) and the World Health Organization (WHO) committed to the introduction of PCVs for infants in Gavi-eligible countries (including PNG). Two PCVs are currently available: i) 10-valent PCV (10vPCV, Synflorix®, GlaxoSmith Kline [GSK], Belgium) containing serotypes 1, 4, 5, 6B, 7F, 9 V, 14, 18C, 19F & 23F; and ii) 13-valent PCV (13vPCV, Prevenar 13®, Pfizer, United States of America [USA]) containing serotypes 3, 6A and 19A in addition to the 10 serotypes in 10vPCV. Both vaccines have been shown to prevent vaccine serotype-associated IPD in high-risk populations [18, 19].

10vPCV also contains non-typeable *Haemophilus influenzae* (NTHi) Protein D as a carrier, which may offer protection against NTHi disease [21]. NTHi was frequently isolated in lung aspirates from children with pneumonia in PNG [22], is a predominant cause of otitis media worldwide [23], and has also been reported as a cause of invasive disease [24–27].

Policy on choice of PCV in low- and middle-income countries has been hampered by the lack of data comparing the two vaccines in high-risk infants. While the three additional serotypes included in 13vPCV improve coverage for prevention of pneumococcal disease, the potential benefits of protection against NTHi-related pneumonia and otitis media by 10vPCV need to be considered [19, 21, 28–30].

The broad range of serotypes causing IPD in PNG and other high-risk populations means that even 13vPCV provides limited coverage (50–70%) against IPD [31–34]. There are also concerns that serotype replacement is more likely to occur in high-risk populations, similar to what was reported for native Alaskan infants after 7vPCV introduction [35].

Despite limited immunogenicity in infants, PPV given to PNG children aged 6 months to 5 years of age has been shown to reduce mortality and severe morbidity due to ALRI [36, 37], with an age-related maturation of IgG responses correlating with efficacy data [38]. Therefore, in high-risk infants, priming with PCV followed by a single dose of PPV in later infancy could potentially increase protection against IPD, particularly for serotypes such as type 2 that is an important cause of meningitis but is not covered by either PCV [31, 39]. This strategy also has the potential to improve duration of protection and reduce the risk of serotype replacement disease compared to PCV alone.

A study in Fiji raised concerns on PPV immunization in young children by reporting that children primed with 7vPCV in infancy followed by a dose of PPV at 12 months of age failed to further boost the already high antibody titres following challenge with 1/5^th^ of a normal PPV dose 5 months later. However, serotype-specific IgG responses in these children did not decline and were robust with high avidity and opsonic functional activity suggesting protective responses [40, 41]. Follow-up of the Fijian study participants at pre-school age showed that serotype-specific antibody levels were similar in those who had and those who had not received PPV at age 12 months, implying that there was no evidence of long-term hyporesponsiveness at pre-school age [42].

In the 7vPCV trial PPV was found to be immunogenic when given at age 9 months to PNG infants previously primed with 3 doses of PCV [5], and antibody titres to PCV and non-PCV serotypes rose in response to a 1/5^th^ PPV challenge dose at age 3–4 years. These data further contribute to the evidence that long-lasting deleterious effects of PPV immunization in infancy are unlikely [43]. Limitations of the 7vPCV trial were the relatively long time interval between receipt of PPV at 9 months and assessment of response to challenge at 3–4 years of age, and that the study did not include a group of children who had received PCV with no PPV. To determine whether priming of high-risk infants with PCV followed by a single dose of PPV in later infancy could be a preferred strategy to increase serotype coverage for IPD, without compromising both short- and long-term protective immune responses, a randomized controlled trial including a control group not receiving PPV is required.

### Rationale for the present study

This head-to-head randomized trial aims to demonstrate that 10vPCV and 13vPCV are suitable vaccines for use under PNG’s accelerated immunization schedule by comparing the safety and immunogenicity of 10vPCV with that of 13vPCV in a 1-2-3-month schedule with and without a PPV booster at 9 months of age. This is important for the following reasons:

- With support from Gavi, an increasing number of low-resource countries are introducing PCV into routine immunization schedules, with a comparable number of 10vPCV and 13vPCV doses being used.
- At the time of the primary schedule of this trial, PCV had not been introduced into PNG and no data were available directly comparing the safety and immunogenicity of these two vaccines in a high-risk population such as PNG. While PNG infants have been receiving 13vPCV since 2014 (after completion of the primary schedule for this trial), it remains important to determine the safety and immunogenicity of 10vPCV should there be a shortage in the supply of 13vPCV or if future research shows that 10vPCV protects against NTHi disease.
- There are limited data on the immunogenicity of PCVs in children with very early onset of dense URT pneumococcal and NTHi carriage.
- The Expanded Program on Immunization (EPI) schedule is accelerated in PNG (ages 1, 2 and 3 months); neither 10vPCV nor 13vPCV13 have been evaluated under this schedule.
- Given the broad range of serotypes causing disease in PNG and in other high-risk populations, it is important to investigate the safety and immunogenicity of a booster dose of PPV at age 9 months following priming with PCV.
- Data are lacking on PCV-induced functional antibody responses and 10vPCV-induced NTHi Protein D antibody responses in populations with high URT carriage rates from a young age.
- While there is evidence that PCV impacts on vaccine serotype-specific URT pneumococcal carriage in high-risk settings [44, 45], there are limited data on the potential impact on carriage density in these settings.

### Study objectives

The overarching aim of the study is to ensure that the PCVs under investigation are suitable for use under PNG’s accelerated immunization schedule, and that a schedule including PPV in later infancy is safe and immunogenic in this high-risk population.

#### The primary objectives of this study are to:

1. Determine the safety and reactogenicity up to 24 months of age of 10vPCV and 13vPCV given in the accelerated 1-2-3-month PNG schedule;
2. Evaluate serotype-specific serum IgG and functional antibody responses at 4 and 9 months of age following completion of 3 doses of 10vPCV or 13vPCV, and antibody persistence and IgG avidity maturation at 23 months of age;
3. Compare serotype-specific serum IgG titres, functional antibody responses and circulating memory B-cells at 10 months of age and the persistence of these responses at 23 months of age in children primed with 10vPCV or 13vPCV who did or did not receive PPV at 9 months of age;
4. Assess the persistence of B-cell memory by measuring circulating vaccine serotype-specific memory B-cells, IgG and opsonophagocytic antibody responses and IgG avidity maturation 1 month after challenge at 23 months with 1/5th of a normal PPV dose in children who did or did not receive PPV at 9 months of age; and
5. Compare the relative impact of 10vPCV and 13vPCV on pneumococcal (total and vaccine type) and NTHi carriage rates and bacterial load up to 2 years of age and the effect of a PPV booster.

#### Secondary objectives of this study are to:

1. Compare development of IgG responses to NTHi Protein D between infants receiving 10vPCV and those receiving 13vPCV; and
2. Describe the frequency of pneumonia and otitis media with perforation associated with pneumococcal vaccine serotypes and/or NTHi isolated from the nasopharynx and/or middle ear discharge.

## Methods

The following paragraphs describe the methods used for enrolment, clinical follow-up and specimen collection and storage.

### Study design and location

Between November 2011 and March 2016, we conducted an open randomized controlled trial of safety and immunogenicity of 13vPCV and 10vPCV given in an accelerated 1-2-3-month schedule, with further randomization of children in each PCV group to receive PPV or no PPV at age 9 months, followed by a 1/5th challenge dose of PPV to all children at age 23 months. The study was conducted in Goroka Town and villages located within an hour’s drive from Goroka in the Eastern Highlands Province of PNG. Goroka is the provincial capital (population ~25,000; altitude 1600 m) and the PNG Institute of Medical Research (PNGIMR) laboratories and clinic are adjacent to Eastern Highlands Provincial Hospital (EHPH), the only tertiary hospital in the area. Outside the urban area, people live in villages often accessible only by 4WD vehicles. Living standards vary widely between urban, peri-urban and rural settings.

### Ethical considerations

Ethical approval was obtained from the PNG Medical Research Advisory Committee (#11.03) and PNGIMR Institutional Review Board (#1028). The study was conducted according to Declaration of Helsinki International Conference on Harmonisation Good Clinical Practice (ICH-GCP) and local ethical guidelines. The study is registered with ClinicalTrials.gov (CTN NCT01619462).

### Recruitment, assent and consent process

Prior to starting the study, field staff visited communities around Goroka Town and surrounding villages to explain the study to community members and seek their willingness to participate in the trial. Pregnant women were invited by research nurses and health extension officers (HEOs) to take part in the study during these field visits or when women attended the PNGIMR clinic. Local drivers who were familiar to and respected by the local communities played an important role in introducing the study team to the communities. Assent was sought from pregnant women and their husbands, contact details were recorded and information sheets were provided. Women who had assented were seen again close to their expected delivery date or when they presented of their own accord at the PNGIMR clinic after delivery. Informed consent was sought at time of enrolment of the child.

#### Inclusion criteria for infants to be enrolled in the study were:

- Aged 28–35 days;
- Resident within 1 h drive from Goroka town; and
- Intending to remain in the study area for at least 2 years.

#### Exclusion criteria were:

- Birth weight < 2000 g;
- Severe congenital abnormality;
- Mother or child known to be positive for Human Immunodeficiency Virus (HIV); or
- Unable to or did not provide consent.

### Study vaccines

A single dose (0.5 mL) of 10vPCV (Synflorix®, GSK, Belgium, batches ASPNA0099AB, ASPNA267DD) contains 1 μg of each pneumococcal polysaccharide for serotypes 1, 5, 6B, 7F, 9 V, 14 and 23F and 3 μg of serotype 4, each conjugated to Protein D of NTHi and 3 μg of serotypes 18C and 19F conjugated to tetanus and diphtheria toxoids, respectively. One dose (0.5 ml) of 13vPCV (Prevenar 13®, Pfizer, USA, batch numbers F36226, G71540) contains 2.2 μg of pneumococcal purified capsular polysaccharides for serotypes 1, 3, 4, 5, 6A, 7F, 9 V, 14, 18C, 19A, 19F and 23F, and 4.4 μg of pneumococcal purified capsular polysaccharides for serotype 6B and 0.125 mg aluminium adjuvant. Each serotype is individually conjugated to non-toxic diphtheria CRM_197_ protein.

Each 0.5 mL dose of PPV (Pneumovax®23 Merck & Co, USA, batch numbers T0861, V1200, K006913) contains 25 μg of purified capsular polysaccharides of each of serotypes 1, 2, 3, 4, 5, 6B, 7F, 8, 9 N, 9 V, 10A, 11A, 12F, 14, 15B, 17F, 18C, 19F, 19A, 20, 22F, 23F and 33F and 0.25% phenol preservative.

### Enrolment, randomization, vaccination and follow-up

Children who met the eligibility criteria were randomized at 1 month of age to receive 10vPCV or 13vPCV using a computer-generated random number list. This assignment was specified inside a sealed envelope with the next sequential number and kept in the participant’s personal folder. Throughout the study laboratory staff was blinded to the group allocation. The vaccine and specimen collection schedules are shown in Table 1. Prior to any vaccination (and PPV challenge at age 23 months), study nurses/HEOs obtained a medical history and examined children to exclude contraindications for vaccination (including temperature ≥ 38 °C, anaphylaxis to previous vaccination, serious neurological illness within 48 h of previous pentavalent vaccine) and specimens were collected. Routine childhood vaccines (including pentavalent whole-cell pertussis, diphtheria, tetanus, Hepatitis B and *Haemophilus influenzae* type B (Hib) vaccine and oral polio vaccine; see Table 1) were administered by the study HEOs/nurses at 1, 2, and 3 months of age, together with the 10vPCV or 13vPCV study vaccines. Information on birth doses of Bacillus Calmette-Guérin (BCG) vaccine and Hepatitis B vaccines administered by hospital staff was collected from hospital records, as were birth weights. At the initial visit mothers were given a follow-up card on which dates for future visits were recorded. Children living in rural areas were generally transported to and from the clinic while urban residents were either brought in or came of their own accord to the clinic following a reminder by mobile phone. At age 4 months children attended the clinic for specimen collection only. At age 6 months, children received their first dose of measles vaccine and a dose of Vitamin A either at the PNGIMR clinic or at other child health clinics. At age 9 months children received a second dose of measles vaccine and Vitamin A, and were randomized within each PCV group to receive or not receive PPV. The PPV group allocation was assigned at enrolment, at the same time as PCV randomization, but kept in an unopened envelope until the 9-month visit. Specimens were collected before vaccination and again 4 weeks later.

**Table 1:**
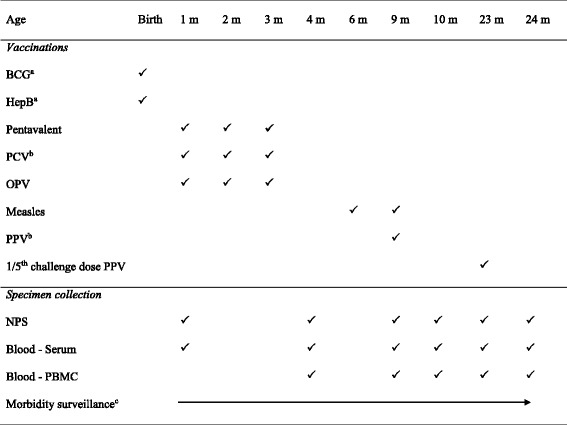
Vaccination and specimen collection schedule

*BCG* Bacillus Calmette-Guérin vaccine; *HepB* Hepatitis B vaccine; Pentavalent is a diphtheria, tetanus, whole cell pertussis, *Haemophilus influenzae* type B, Hepatitis B combination vaccine; *PCV* 10-valent or 13-valent pneumococcal conjugate vaccine; *OPV* oral polio vaccine; *PPV* 23-valent pneumococcal polysaccharide vaccine; *NPS* nasopharyngeal swab; *PBMC* peripheral blood mononuclear cells

^a^Birth doses of BCG and Hepatitis B were given by hospital nurses before enrolment of infants into the study

^b^PCV and PPV are study vaccines while the other vaccines were routine EPI vaccines in PNG at the time of the trial

^c^Morbidity surveillance was conducted throughout the duration of the trial. Vitamin A was given at 6 and 9 months of age

All participants were given a 1/5^th^ challenge dose of PPV at age 23 months with specimens collected immediately before the challenge and 4 weeks later. All vaccinations were documented in the child’s health book held by the mother/guardian and also written on the child’s follow-up card, which was stored electronically. Photocopies of records in the parent-held books (which include information on any illness episode) were stored in the child’s personal folder.

Every effort was made to locate participants for follow-up in a timely manner. Between 10 and 23 months, nurses and field staff attempted to contact participants’ caregivers at least twice to find out about any intervening illness and changes in address. Children were withdrawn from the study if the child could not be located on 3 home visits or information had been obtained from relatives that the family had moved out of the study area permanently, or if parents withdrew their consent for continuation in the study. Children were also withdrawn by the investigators following a protocol violation.

### Monitoring for adverse reactions

Following each vaccination, children were observed at the clinic for 1 h for immediate local and systemic reactions and generally taken home by the study team. This helped locate participants for subsequent visits. Study nurses/HEOs visited children at home 24–48 h post-vaccination to assess local or systemic side effects and they provided treatment as required.

### Specimen collection

A nasopharyngeal swab (NPS) to assess pneumococcal and NTHi carriage and bacterial load was collected at ages 1, 4, 9, 10, 23 and 24 months (Table 1) as previously described [8] except that a flocked swab (Copan Diagnostics, USA) was used. NPS samples were placed in skim-milk-tryptone-glucose-glycerol broth (STGGB) and stored at −80 °C within 2 h of collection. Culture and pneumococcal serotyping were conducted at PNGIMR as previously described [8]. Aliquots from the stored NPS were sent in liquid nitrogen to the University of Western Australia to measure bacterial load. If middle ear discharge was seen, a flocked swab was used to collect and store the purulent discharge in STGGB for culture, identification and characterisation of bacterial pathogens [8].

Venous blood samples (up to 3 mL) for *serum* collection were collected at ages 1, 4, 9, 10, 23 and 24 months (Table 1) to measure pneumococcal serotype-specific IgG antibody titres [5], IgG avidity [46], opsonophagocytic antibody titres [47], and NTHi Protein D-specific IgG antibody levels [48]. No more than 3 attempts to collect blood were undertaken at a single time point. On occasions when parents agreed, a further attempt at blood collection was undertaken a few days later. Serum samples were aliquoted and stored at −80 °C. Serotype-specific IgG antibody responses were measured at PNGIMR using a WHO standardized pneumococcal enzyme-linked immunosorbent assay (ELISA) [49]. Serum aliquots were sent in liquid nitrogen to the University of Western Australia (UWA) to conduct functional pneumococcal antibody assays and NTHi protein D antibody assays.

From age 4 months onwards, at each routine follow-up visit, we aimed to collect 2–7 mL of heparinized venous blood samples to isolate peripheral blood mononuclear cells (PBMCs) to measure pneumococcal serotype-specific B-cell responses and pneumococcal protein-specific T-cell responses as shown in Table 1 [50]. After immediate processing at PNGIMR, PBMCs were cryopreserved and sent in batches in liquid nitrogen vapour phase to UWA.

Detailed laboratory methods will be reported elsewhere with laboratory results.

### Morbidity surveillance

Passive morbidity surveillance was conducted. Parents were encouraged to attend the PNGIMR clinic if their child was unwell. Generally parents liked to bring their children directly to the PNGIMR clinic as they were seen faster than at routine outpatient clinics, they knew the staff, and were assisted with transport if required. In addition, family members other than the study child could be seen at the PNGIMR clinic for the duration of the study. Children were referred to pediatricians at EHPH as required. The EHPH pediatric ward was also monitored for any hospitalizations of study participants. Information on any intervening illnesses was documented on a morbidity report form and in the parent-held child’s health book. Children were treated according to standard management protocols in PNG [51].

ALRI was defined as mild (cough and raised respiratory rate, namely >60/min in infants less than 2 months of age, or >40/min in infants aged 2 months and older), moderate (as for mild with chest wall in-drawing), or severe (as for moderate with cyanosis or inability to feed or restlessness or heart failure). This is a slight modification from that used generally in PNG. Standard treatment protocols in PNG recommend hospitalization for all moderate or severe ALRIs.

A serious adverse event (SAE) was defined as any event requiring hospitalization or resulting in death. In 2015 there were disruptions of EHPH services. During this period an SAE was also documented for illnesses (mainly moderate ALRI cases) deemed serious enough to require hospitalization, but the children had to be treated as outpatients and were monitored closely. Blood cultures and NPS were collected when children were seen with a temperature > 38 °C or a provisional diagnosis of moderate or severe ALRI, or meningitis.

### Outcomes

#### PCV outcome measures are:

1. Safety and reactogenicity of 10vPCV and 13vPCV in PNG infants;
2. Serum PCV serotype-specific IgG and opsonizing antibody geometric mean titres (GMTs), and proportions of children with serum serotype-specific IgG antibody concentrations ≥0.35 μg/mL and opsonizing titres ≥8 for PCV serotypes at 4 and 9 months of age;
3. Serum *H. influenzae* Protein D IgG GMTs at 4 and 9 months of age;
4. Number of circulating memory B-cells specific for PCV serotypes and PCV carrier proteins (NTHi Protein D, Tetanus Toxoid and CRM_197_) and PCV serotype-specific IgG avidity maturation at 4 and 9 months of age;
5. T-cell responses to PCV carrier proteins (NTHi Protein D, Tetanus Toxoid and CRM_197_) at 4 and 9 months of age;
6. Nasopharyngeal carriage rates of *S. pneumoniae* (vaccine and non-vaccine serotypes) and serotypeable and non-serotypeable *H. influenzae* at 1, 4 and 9 months of age;
7. Pneumococcal and *H. influenzae* nasopharyngeal density at 1, 4 and 9 months of age; and
8. Rates for hospitalization with ALRI or record of otitis media with perforation between 4 and 9 months of age.

#### PPV outcome measures are:

1. Serum PCV and PPV serotype-specific IgG and opsonizing antibody GMTs, and proportions of children with PCV and PPV serotype-specific IgG concentrations ≥0.35 μg/mL and opsonizing titres ≥8 at 10 and 23 months of age;
2. Number of circulating memory B-cells and IgG avidity maturation for PCV and non-PCV serotypes included in PPV at 10, 23 and 24 months of age;
3. Nasopharyngeal carriage rates and density of serotype-specific *S. pneumoniae*, and serotypeable and non-serotypeable *H. influenzae* at 10, 23 and 24 months of age; and
4. Rates for hospitalization with ALRI or record of otitis media with perforation between 10 and 24 months of age.

### Study and data management

To ensure any problems or queries were addressed as soon as possible, investigators in PNG and Australia attended monthly teleconferences.All data collection forms were checked manually before entry into a password-protected computer file using FileMaker Pro version 11 (Santa Clara, CA, USA).

### Data safety monitoring board

A data safety monitoring board (DSMB) was established, which included independent clinicians in PNG and Australia. All SAEs were reported within 24 h to an appointed Papua New Guinean Safety Monitor and the Chair of the DSMB for review, followed by a final report with outcome. The Chair of the DSMB was informed immediately when a death occurred. A quarterly report was prepared by the investigators and sent to the Chair of the DSMB and distributed to other DSMB members. This included a summary of all serious adverse events, all morbidities, protocol violations, and status of enrolment and follow-up. A teleconference with DSMB members and the study investigators was held annually.

### Sample size calculations

Sample size calculations were based on an anticipated 80% follow-up rate with evaluable samples to the final visit based on results from our 7vPCV trial (87% at age 18 months) [5]. Based on the study’s primary outcome, it was postulated that 90% of children would achieve serotype-specific antibody concentrations ≥0.35 μg/ml at 4 months of age for serotypes included in the PCV children were given. Based on this assumption, a sample size of 100 children (receiving 10vPCV or 13vPCV) would give us 95% confidence that the true proportion is within 6% of this level of 90%. For bacterial load analysis, previous data for *H. influenzae* bacterial loads [52] indicate that a within-group standard deviation in log_10_ bacterial load (copies/ml) of 2.0 can be expected. With a sample size of 50 in each group (after randomization into PCV and PPV group), this would give 80% power to detect a between-group difference in mean log_10_ bacterial load of 0.9. In view of the higher loss to follow-up than anticipated during the first year of the study, the total number of children to be enrolled was increased from 200 to 260.

### Statistical methods

Statistical analysis is by intention to treat. All serum antibody concentrations and opsonophagocytic titres were log-transformed and geometric mean concentrations (GMC) and geometric mean titres (GMT) calculated respectively with 95% confidence intervals. To compare continuous and categorical variables between groups, Mann-Whitney tests and Pearson chi-square tests were used respectively. Spearman rank correlation analysis was used to investigate correlations between serotype-specific opsonophagocytic and IgG antibody concentrations. Linear and logistic regression models were used for further analyses examining associations between specific outcomes and different factors incorporating generalised estimating equations to take into account multiple samples from individual children. For all analyses, test outcomes were considered to be significant if the *p*-value is smaller or equal to 0.05.

## Results

### Assent and enrolment

**Fig. 1:**
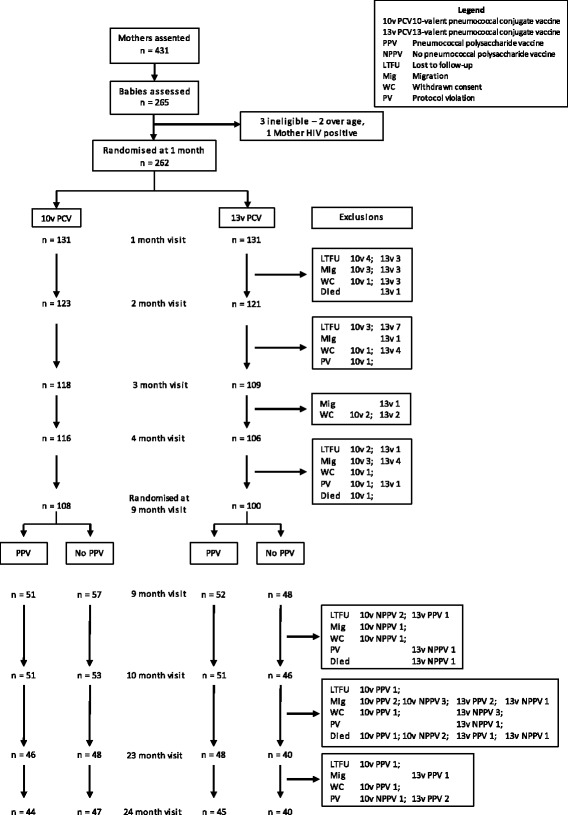
flow chart

A total of 431 women assented to having their child participate in the study, of which 265 (61%) gave their informed consent. Three children did not meet inclusion criteria (2 were overage and 1 child’s mother was HIV-positive). Hence 262 children were formally enrolled into the study between 14 November 2011 and 16 April 2014, including 131 children randomized to receive 10vPCV and 131 randomized to receive 13vPCV. A flow chart (Fig. 1) shows the number of women who assented, number of infants randomized to receive one or other PCV at 1 month of age, number of children seen at subsequent visits, number of withdrawals and reasons for withdrawal according to randomization schedule.

### Withdrawals

A total of 86 (33%) children were withdrawn from the study (including deaths and protocol violations), the majority before age 9 months (41% before age 3-month visit, 63% before 9-month visits) (Fig. 1). Parents withdrew their consent for 20 children (8%) (8 in 10vPCV group and 12 in 13vPCV group); 25 children (9.5%) migrated with their parents to another location (12 in 10vPCV group and 13 in 13vPCV group); and 25 children (9.5%) were recorded as lost to follow-up after at least 3 unsuccessful home visits by field staff (13 in 10vPCV group and 12 in 13vPCV group). Of the children lost to follow up, two-thirds could not be located within 2 months of enrolment, while approximately one-third of those who migrated out of the study area did so before age 4 months. Approximately two-thirds of parents who withdrew consent did so before the 4-month visit. None of the withdrawals were related to an adverse event.

There were 7 deaths, none of which were related to any vaccination (4 in the 10vPCV group and 3 in the 13vPCV group). Documented causes of death, age at death and PCV/PPV status were as follows:

- Severe pneumonia and presumed pulmonary tuberculosis; 9 months; 13vPCV
- Blood dyscrasia, anaemia; 9 months; 10vPCV
- Moderate pneumonia, malnutrition, gastroenteritis; 15 months; 10vPCV, no PPV
- Malnutrition, anaemia, presumed pulmonary tuberculosis; 17 months; 10vPCV, no PPV
- Gastroenteritis with dehydration; 17 month; 13vPCV, PPV
- Gastroenteritis with severe dehydration; 18 months; 10vPCV, PPV
- Severe malnutrition, dysentery, anaemia; 19 months; 13vPCV, no PPV

One other child received the first dose of 13vPCV but soon after was diagnosed with a retroperitoneal tumour, at which time this child was withdrawn from the study and died at age 2 months.

There were 8 protocol violations: 3 were related to the PCV schedule (2 in the 10vPCV group, 1 in the 13vPCV group), 1 was related to PPV immunization (within the 13vPCV group) and 4 were related to the low-dose challenge at 23 months of age (1 in the 10vPCV/no PPV group, 2 in the 13vPCV/no PPV group and 1 in the 13vPCV/PPV group).

### Follow-up of study participants

Of the 262 children enrolled in the study who received PCV at age 1 month, 93% received 2 doses of PCV and 87% received 3 doses of PCV. At age 9 months 79% of enrolled children were randomized to receive PPV or no PPV; 77% of the 262 children were seen at the 10 month visit; 69% at 23 months; and 67% completed the study at 24 months of age with appropriate immunization and challenge. Follow-up was similar for the different PCV/PPV groups.

Table 2 shows that children received PCV and PPV in a timely manner with no difference in age at vaccination and follow-up between groups. The table also shows the gender distribution and average body weight according to PCV/PPV group at different time points. There were no differences between the groups.

**Table 2:** Description of study population according to randomization groups

	10vPCV		13vPCV	
Number enrolled	131		131	
Male, number (%) at enrolment	66 (50%)		72 (55%)	
Age (days) at study visits				
Visit 1 m/PCV1, mean ± sd	30.9 ± 2.5 (*n* = 131)		31.0 ± 2.2 (*n* = 131)	
(Min-Max)	(25–39)		(27–40)	
Visit 2 m/PCV2, mean ± sd	61.6 ± 3.4 (*n* = 122)		61.9 ± 4.0 (*n* = 121)	
(Min-Max)	(55–77)		(56–84)	
Visit 3 m/PCV3, mean ± sd	94.0 ± 7.8 (*n* = 119)		92.5 ± 4.4 (*n* = 109)	
(Min-Max)	(84–134)		(84–112)	
Weight (kg)				
Birth weight, mean ± sd	3.4 ± (*n* = 97)		3.3 ± (*n* = 105)	
(Min-Max)	(2.2–4.5)		(2.3–4.5)	
Visit 1 m, mean ± sd	4.4 ± 0.7 (*n* = 127)		4.3 ± 0.5 (*n* = 126)	
(Min-Max)	(2.5–6.1)		(2.7–5.7)	
Visit 2 m, mean ± sd	5.3 ± 0.7 (*n* = 122)		5.3 ± 0.7 (*n* = 120)	
(Min-Max)	(3.4–6.8)		(2.9–7.2)	
Visit 3 m, mean ± sd	6.1 ± 0.9 (*n* = 114)		6.0 ± 0.8 (*n* = 108)	
(Min-Max)	(4.0–8.5)		(4.1–8.1)	
	10vPCV		13vPCV	
	No PPV	PPV	No PPV	PPV
Number followed-up at 9 months	57	51	48	52
Male, number (%) at 9 months	29 (51%)	24 (43%)	26 (54%)	29 (54%)
Age (months)				
Visit 9 m, mean ± sd	9.2 ± 0.7 (*n* = 57)	9.5 ± 1.2 (*n* = 51)	9.06 ± 0.2 (*n* = 48)	9.2 ± 0.5 (*n* = 52)
(Min-Max)	(8.2–13.1)	(8.9–14.4)	(8.6–9.9)	(8.9–11.9)
Visit 23 m, mean ± sd	23.4 ± 0.8 (*n* = 47)	23.3 ± 0.6 (*n* = 46)	23.3 ± 0.8 (*n* = 40)	23.3 ± 0.7 (*n* = 46)
(Min-Max)	(22.1–27.1)	(22.9–26.3)	(22.9–26.5)	(22.6–26.0)
Visit 24 m, mean ± sd	24.5 ± 1.0 (*n* = 47)	24.6 ± 0.9 (*n* = 44)	24.4 ± 0.8 (*n* = 40)	24.5 ± 1.1 (*n* = 45)
(Min-Max)	(23.2–28.1)	(23.9–27.9)	(23.9–30.1)	(23.7–30.1)
Weight (kg)				
Visit 9 m, mean ± sd	8.4 ± 1.1 (*n* = 53)	8.1 ± 1.3 (*n* = 51)	8.1 ± 0.8 (*n* = 45)	8.0 ± 0.9 (*n* = 52)
(Min-Max)	(6.5–11.2)	(6.3–14.0)	(6.5–10.4)	(6.6–10.5)
Visit 23 m, mean ± sd	11.3 ± 1.2 (*n* = 44)	10.7 ± 1.4 (*n* = 42)	10.9 ± 1.3 (*n* = 38)	11.0 ± 1.5 (*n* = 37)
(Min-Max)	(9.7–14.0)	(7.7–14.0)	(8.8–14.0)	(8.9–15.0)
Visit 24 m, mean ± sd	11.7 ± 1.5 (*n* = 44)	11.3 ± 1.4 (*n* = 40)	11.3 ± 1.2 (*n* = 35)	11.17 ± 1.4 (*n* = 42)
(Min-Max)	(8.9–15.0)	(8.1–14.0)	(9.0–14.0)	(9.0–15.0)

### Specimen collection

NPS and venous blood samples for serum collection were successfully obtained from more than 96% of attendees at different ages, while PBMCs were successfully isolated from 64 to 69% of blood samples (Table 3). On 4 occasions parents declined blood collection from their children.

**Table 3:** Number of collected specimens (% of participants seen at specified age), blood volumes and PBMCs^a^ isolated at study visits

Age	1 month	4 months	9 months	10 months	23 months	24 months
Study participants^b^	262	222	208	201	182	176
Nasopharyngeal swabs	262 (100%)	215 (97%)	205 (99%)	199 (99%)	182 (100%)	175 (99%)
Blood (serum)	262 (100%)	213 (96%)	201 (97%)	196 (98%)	182 (100%)	175 (99%)
*Mean volume (mL)*	1.8	2.3	2.3	2.2	2.4	2.3
*(Min-Max)*	(0.1–5.0)	(0.2–5.0)	(0.2–5.0)	(0.1–4.0)	(0.5–4.0)	(0.3–4.0)
Blood (PBMCs^a^)	–	142 (64%)	143 (69%)	128 (64%)	126 (69%)	119 (68%)
*Mean volume (mL)*	–	1.9	2.0	1.9	1.9	1.8
*(Min-Max)*		(0.1–4.5)	(0.5–4.0)	(0.4–4)	(0.3–4.0)	(0.3–3.0)
*Mean number of cells ×10* ^*6*^ *(95% CI)*		8.5 (7.3–9.6)	10.1 (8.8–11.4)	9.4 (8.3–10.4)	8.7 (7.5–9.8)	8.8 (7.7–10.0)

^a^Peripheral blood mononuclear cells ^b^Numbers exclude those who did not meet inclusion criteria and those who were lost to follow-up or withdrawn

## Discussion

To our knowledge, our study is the only trial directly comparing safety and immunogenicity of two different PCVs followed by a PPV booster dose. A trial in The Gambia directly compared safety and immunogenicity of 10vPCV and 13vPCV as part of a larger trial of new experimental pneumococcal protein-based vaccines, but did not include a PPV booster [53]. Two other trials comparing different schedules of the two PCVs are underway, one in Vietnam (NCT01953510) and another in Australian Aboriginal children (ACTRN12610000544077 and NCT01174849).

### Study strengths and limitations

Despite logistical difficulties in conducting the study in urban and rural areas of the Eastern Highlands Province of PNG, a large enough number of samples were successfully collected to address study outcomes. Two-thirds of those enrolled in this study were fully followed up to 2 years of age, with sera and NPS collected at >1200 routine visits and high numbers of PBMCs (Table 3) isolated from 658 blood samples from age 4 months onwards. This is a reflection of the long-established relationship and trust between the community and PNGIMR staff, in addition to the perseverance of field staff in locating participants.

The two-stage process of assent followed by formal consent was important, allowing parents to discuss their participation with family members. Providing medical treatment to participants and their families and close follow-up in the event of illness was appreciated and a motivation to take part in the study. In addition, all study children received hand-knitted sweaters donated by Australian volunteers, and at the end of the study parents received a certificate of participation with a photograph of their child.

Every vaccination entailed at least three trips to communities (pick-up and drop-off, and 24–48 h follow-up visit), with additional visits needed if not located the first time. Roads were at times impassable and locals repairing roads sometimes charged a ‘toll’. In 2012 there were national elections and some families returned to their home villages outside the study area to vote and did not return. Furthermore, disputes between supporters of rival candidates at times restricted movement in the study area.

Despite every effort, approximately one-third of participants did not complete the study. This is in part the result of increasing mobility of the population: 10% of participants migrated out of the area during this study compared with 5% during the earlier 7vPCV study conducted between 2005 and 2009 [54]. Loss to follow-up and withdrawal of consent tended to occur at a young age, similar to the 7vPCV study [54]. In some cases, husbands had not been part of the assent and consent processes and subsequently indicated they did not want their children to participate in the study. While every effort was made to provide full information about what the study entailed, mothers may on further reflection have had doubts about taking part in the study. Some parents were concerned about the volume of blood that was being collected from their child. Collecting blood samples from young children was sometimes difficult, though the nurses/HEOs did not persevere if the child or parent was distressed.

Having a Papua New Guinean doctor to oversee the clinical activities and train nurses and HEOs was a great advantage and much appreciated by parents. When the study doctor left a year and a half into the study to pursue pediatric training, a succession of three competent HEOs oversaw the field and clinical activities. However, these staff changes reduced continuity over the course of the study.

In contrast to laboratory staff who were blinded to the vaccine randomization, study nurses were not blinded due to the limited number of nurses/HEOs available to administer vaccines and conduct follow-ups. However, whenever possible, different nurse/HEOs administered vaccines to those assessing reactogenicity. It is acknowledged that this could potentially impact on the interpretation of clinical outcomes, but cannot affect the primary immunogenicity outcomes.

Despite the launch of the 13vPCV program in PNG in 2014, vaccine was not available in the EHP until 2015 and has been introduced slowly since: coverage with one dose of 13vPCV was only around 25% and with three doses around 5% in late 2015 in the study area [55]. Herd protection following launch of the PNG 13vPCV program in PNG in late 2014 is therefore unlikely to have impacted on the results of the present study.

Notably, there were no deaths among study participants due to uncomplicated pneumonia in a country where pneumonia remains the most common cause of childhood death [3]. This is attributed to all study children receiving both pneumococcal and Hib vaccines and close monitoring of study participants when they were sick. While morbidity among study participants will be reported fully elsewhere, 2 of the 3 cases of culture-proven pneumococcal bacteraemia were due to non-PCV serotypes and the third child with a PCV serotype-related bacteraemia had only received one dose of PCV at the time of death [56]. The mean age at death was older than previously recorded [57], with 5 of the 8 deaths occurring in the second year of life. While a diagnosis of pulmonary tuberculosis was assigned for two children who died, these were not laboratory-confirmed. The emergence of malnutrition as an important cause of death in PNG has been highlighted elsewhere [58]. This may be related to undiagnosed tuberculosis, which was previously rare in the highlands of PNG, overall poverty, or a reflection of societal challenges including unwanted pregnancies [59, 60] and associated neglect of children [61]. Finally, infants with a birth weight of less than 2000 g were excluded as low birth weight was used as a surrogate marker for prematurity, which may affect immune responses to vaccines including PCVs [62], as well as that  prematurity is hard to assess in this setting. As is true for randomized clinical trials in general, field studies conducted post-implementation may find different results in individuals due to the influence of various external factors. However, the authors believe that the results for 10vPCV and 13vPCV immunogenicity and safety are largely generalizable to newborns in PNG and other settings where pneumococcal infections are highly endemic.

## Conclusions

The introduction of PCV in high-risk settings facilitated by Gavi is likely to have a significant impact on the burden of IPD, although the high proportion of pneumococcal disease due to non-vaccine serotypes raises concerns with regard to serotype replacement. The lack of data comparing PCVs has made it difficult for countries to choose the PCV likely to provide the most benefit. This study provides direct comparison of safety, immunogenicity (including functional antibody responses) and impact on carriage rates and bacterial load in a randomized controlled trial of an accelerated 3-dose schedule of 10vPCV and 13vPCV in a low-income setting. Following completion of the primary schedule in this trial without immediate concerns regarding reactogenicity and safety of either PCV, 13vPCV was introduced into the PNG EPI Program in 2014. In addition to comparison of currently available PCVs, this study will provide evidence on the potential advantages of PPV following PCV immunization in infancy in high-risk settings, including immune protection against important IPD-causing non-PCV serotypes [31, 34], boosting of immune responses against PCV serotypes to enhance long-term immunity, and will directly investigate any effects on B-cell memory. Samples collected in this cohort are currently undergoing laboratory testing to determine the impact of the different vaccine schedules and formulations on nasopharyngeal carriage, antibody production and function, and immune memory response. Assessment of antibody and T-cell responses to pneumococcal and NTHi proteins will aid an understanding of the development of natural immunity to these antigens relevant to development of protein-based vaccines. These studies will provide data to inform policy on pneumococcal vaccine schedules in countries with children at high risk of invasive pneumococcal disease.

## Abbreviations

10vPCV: 10-valent pneumococcal conjugate vaccine
13vPCV: 13-valent pneumococcal conjugate vaccine
AE: Adverse event
ALRI: Acute lower respiratory infection
BCG: Bacillus Calmette-Guérin
CSL: Commonwealth Serum Laboratories Ltd.
DSMB: Data safety monitoring board
EHPH: Eastern Highlands Provincial Hospital
ELISA: Enzyme-linked immunosorbent assay
EPI: Expanded Program on Immunization
Gavi: Global Alliance for Vaccines and Immunization
GMC: Geometric mean concentration
GMT: Geometric mean titre
HEO: Health extension officer
Hib: *Haemophilus influenzae* type B
HIV: Human Immunodeficiency Virus
ICH-GCP: International Conference on Harmonisation Good Clinical Practice
IPD: Invasive pneumococcal disease
NHMRC: National Health and Medical Research Council
NPS: Nasopharyngeal swab
NTHi: Non-typeable *Haemophilus influenzae*
PBMC: Peripheral blood mononuclear cells
PCV: Pneumococcal conjugate vaccine
PNG: Papua New Guinea
PNGIMR: PNG Institute of Medical Research
PPV: Pneumococcal polysaccharide vaccine
SAE: Serious adverse event
STGGB: Skim milk tryptone-glucose-glycerol broth
URT: Upper respiratory tract
UWA: University of Western Australia
WHO: Word Health Organization
